# 聚二甲基硅氧烷微流控适配体传感器：开启生物标志物即时检验新时代

**DOI:** 10.3724/SP.J.1123.2025.10003

**Published:** 2026-05-08

**Authors:** Yuanchao YANG, Renfang XIAO, Yangtao WU, Li BAI, Xingya WANG, Jingbo ZHAI

**Affiliations:** 1.内蒙古民族大学基础医学院，内蒙古 通辽 028000; 1. School of Basic Medical Sciences，Inner Mongolia Minzu University，Tongliao 028000，China; 2.内蒙古民族大学蒙医药学院，内蒙古 通辽 028000; 2. Mongolia Medical College，Inner Mongolia Minzu University，Tongliao 028000，China; 3.人兽共患病防控自治区高等学校重点实验室，内蒙古 通辽 028000; 3. Key Laboratory of Zoonose Prevention and Control at Universities of Inner Mongolia Autonomous Region，Tongliao 028000，China; 4.内蒙古自治区 布鲁氏菌病防治工程技术研究中心，内蒙古 通辽 028000; 4. Brucellosis Prevention and Treatment Engineering Research Center of Inner Mongolia Autonomous Region，Tongliao 028000，China

**Keywords:** 微流控芯片, 适配体传感器, 聚二甲基硅氧烷, 即时检验, 生物标志物, microfluidic chip, aptasensor, polydimethylsiloxane （PDMS）, point-of-care testing （POCT）, biomarker

## Abstract

在微流控芯片合成材料中，聚二甲基硅氧烷（PDMS）作为一种聚合物材料，因制造工艺简便、透明度高、化学稳定性好及具有生物相容性等特性，已成为构建微流控芯片的首选材料。将适配体作为特异性生物受体整合于PDMS微流控芯片构建的生物传感器，即PDMS微流控适配体传感器，其核心优势在于融合了适配体这一独特的生物识别元件与微流控技术。微流控技术可将生物分子间的相互作用转化为便于处理和报告的可读信号，从而提供具备高特异性与高灵敏度的传感手段，极大地推动了生物标志物即时检验（POCT）的发展，可广泛应用于癌症筛查、病原体检测等，实现了检测的快速性、准确性与便携性。PDMS微流控适配体传感器的特点为成本低、消耗少、耗时短、可抛弃等，在POCT领域可有效减少样品和试剂的消耗并缩短检测时间。同时，通过优化微流控芯片微通道设计、适配体固定化方法以及信号放大策略等可进一步提升PDMS微流控适配体传感器的性能，拓宽其检测范围。本文对微流控技术的定义与发展、PDMS材料及制备工艺在微流控芯片制造中的应用研究及PDMS微流控适配体传感器的开发等进行了详细阐述，同时列举了光学、电化学和基于双重检测模式的双模态PDMS微流控适配体传感器在生物标志物POCT领域的应用，为未来新型微流控适配体传感器的开发及应用提供了理论支持。

## Abstract (en)

Microfluidic technology， an emerging micro-nano technology， facilitates the precise manipulation of trace liquids via diverse microstructures. It is distinguished by its miniaturization and cost-effectiveness. Among the synthetic materials employed for microfluidic chips， polydimethylsiloxane （PDMS）， a polymer material， has emerged as the optimal choice for fabricating microfluidic chips. This preference stems from its straightforward manufacturing process， high transparency， outstanding chemical stability， and biocompatibility. Biosensors developed by integrating aptamers， which serve as specific biological receptors， into PDMS microfluidic chips are termed PDMS microfluidic aptasensors. These aptasensors capitalize on the distinctive advantages of aptamers as biological recognition elements in conjunction with microfluidic technology. Microfluidic technology transforms the interactions between biomolecules into readable signals that are readily processed and reported， thereby offering sensing methods characterized by high specificity and sensitivity. This advancement significantly propels the development of point-of-care testing （POCT） for biomarkers， enabling extensive applications in areas such as cancer screening and pathogen detection， and achieving rapid， accurate， and portable testing. The attributes of PDMS microfluidic aptasensors encompass low cost， minimal consumption， short processing time， and disposability. These features effectively curtail the consumption of samples and reagents and shorten the testing duration in the POCT realm. Moreover， optimizing microchannel designs， aptamer immobilization methods， and signal amplification strategies can further enhance the performance of PDMS microfluidic aptasensors and expand their detection range. This paper elaborately expounds on the definition and development of microfluidic technology， the application research of PDMS materials and their preparation processes in microfluidic chip manufacturing， as well as the development and processing of PDMS microfluidic aptasensors. It also enumerates the applications of optical， electrochemical， and dual-mode PDMS microfluidic aptasensors based on dual detection modes in the field of biomarker POCT， thus providing theoretical support for the future development and application of novel microfluidic aptasensors.

生物标志物在人类生命健康领域扮演着至关重要的角色^［1］^。从分子水平到细胞水平，生物标志物在疾病早期诊断、分类、治疗效果监测、预后评估以及健康体检等方面均发挥着重要作用^［2，3］^。传统的生物标志物检测技术普遍存在一定的局限性，例如细菌分离培养技术耗时较长、部分细菌培养难度较大且存在泄漏风险；免疫学（血清学）检测灵敏度欠佳、易出现交叉反应；聚合酶链式反应（polymerase chain reaction， PCR）技术依赖大型实验仪器、专业实验试剂以及专业人员操作等，均难以满足现代医疗对快速、准确的即时检测（point-of-care testing， POCT）技术的需求。因此，亟需研发一种创新性的检测技术，以实现生物标志物的现场精准检测。

微流控技术（microfluidics）作为一门新兴的综合性技术，目的是在微米（μm）尺度的微小腔室芯片内，对微升（μL）至皮升（pL）的微小体积流体进行精准的输送、混合、分离及其他操控或处理^［4］^，为研究领域带来了集成化、小型化和自动化的优势^［5］^。1990年，Manz等^［6］^首次提出微流控技术。微流控芯片在生化、生物医学、疾病诊断、药物设计和制药等领域具有多方面优势，例如高通量筛选、减少样品与试剂用量、精准控制反应时间、可穿戴及现场分析等^［7-9］^。微流控芯片将常规化学、生物学等领域涉及的多个基本操作单元集成于几平方厘米的芯片之上，由微尺度通道（微通道）构建成网络，借助可控流体贯穿整个系统，实现常规化学或生物实验室的检测功能，其被称作“芯片实验室”（lab-on-a-chip， LOC）^［10］^。在微流控芯片的合成过程中，聚合物材料因具有应用范围广泛、种类繁多、易于加工等特性，成为首选的合成材料。其中的高分子材料聚二甲基硅氧烷（polydimethylsiloxane， PDMS）凭借其自身较高的理化稳定性、良好的生物相容性等优势，被广泛应用于合成检测生物标志物的微流控芯片。近年来，基于适配体（aptamer， Apt）研发的生物传感器——适配体传感器受到了研究人员的关注。适配体是一段单链寡核苷酸序列，通过指数富集的配体系统进化技术（systematic evolution of ligands by exponential enrichment， SELEX）从体外化学合成的单链寡核苷酸文库中筛选获得^［11，12］^。适配体能够与特定靶标高亲和、高特异结合，其作用机制与抗原-抗体间的相互作用类似，故而亦被称为“化学抗体”或“人工抗体”^［13］^。适配体作为单链寡核苷酸，相较于传统抗体具备更高的选择性、亲和力以及良好的稳定性（图1）^［14］^。此外，适配体具备易于修饰的特性，能够保障其与检测条件实现相互兼容^［15，16］^。当前，基于适配体研发的适配体传感器已在医疗诊断^［17，18］^、食品安全^［19，20］^、环境监测^［21，22］^等领域获得广泛应用。适配体传感器主要借助不同类型的生物传感技术开展检测工作，能够在生物医学检测领域精准识别特定的细胞^［23］^、细菌^［24］^、蛋白质分子^［18］^等生物标志物。将微流控芯片与适配体传感器相结合研发的微流控适配体传感器（microfluidic aptasensor），融合了微流控技术的微型化特点以及适配体传感器的高特异性与高亲和力，成为适配体传感器领域的一个新兴分支。微流控的微型化特征使适配体传感器具备在极小空间内高效捕获并检测靶标的能力，提升了检测的灵敏度与特异性。对于传统检测手段难以识别的微量物质，也可借助微流控适配体传感器实现精准检测，彰显了该技术的优越性。

**图1:**
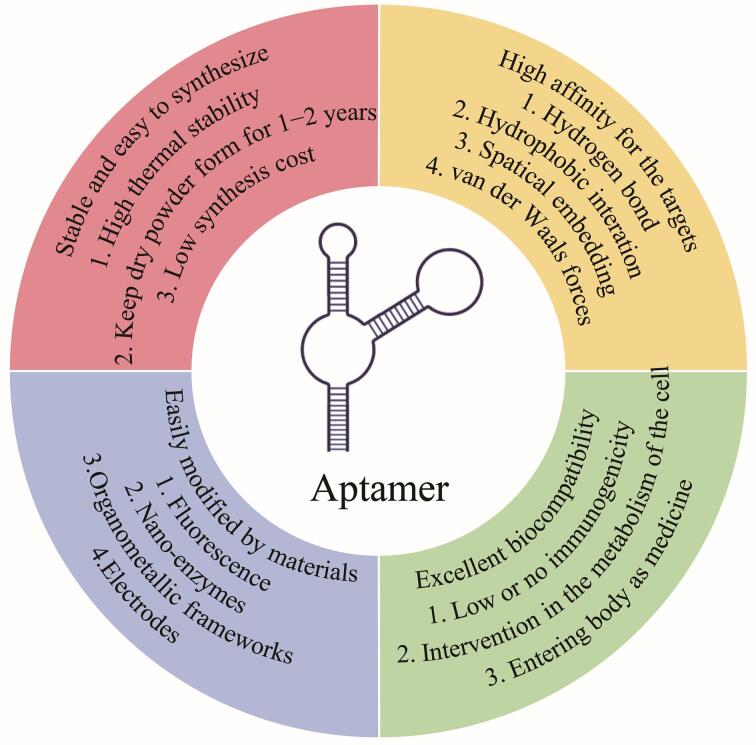
适配体特性

本文系统地阐述了PDMS材质及其制造工艺在芯片合成中的应用，以及PDMS微流控适配体传感器的开发与处理，同时还深入剖析了基于PDMS微流控芯片开发的光学、电化学及双模态微流控适配体传感器在生物标志物POCT领域的应用，旨在为未来新型PDMS微流控适配体传感器的研发与应用提供具有重要价值的参考。

## 1 PDMS微流控芯片的化学合成

PDMS微流控芯片的化学合成是构建生物标志物高效检测平台的关键环节。在芯片的合成与制备方法方面，PDMS凭借其独特的物理化学特性成为一种理想的材料。通过精准调控各个步骤的参数，能够制备出具有良好光学透明性、弹性和生物相容性的PDMS微流控芯片，为后续的微流控操作提供可靠保障。

### 1.1 芯片合成材料及制备工艺

微流控芯片应用的核心在于对其自身合成材料的精准筛选及相应制备工艺的优化。微流控技术的起源与微电子学领域存在紧密关联，半导体材料晶体硅（Si）成为微流控芯片开发领域中最早被应用的材料，随后被玻璃取代^［5，25］^。玻璃具有化学稳定性、机械稳定性、光学透明性、良好的生物相容性以及微米级通道蚀刻能力，这使其成为生物学研究的理想材料。随着科学技术的发展，新兴的聚合物材料凭借应用范围广泛、种类繁多、性价比高、易于加工以及良好的可扩展性等特性，在新型材料中崭露头角，并逐渐取代玻璃成为合成芯片的首选材料。聚合物材料呈现出与玻璃相近的理化特性，同时具备耐热性、耐寒性、耐腐蚀性等稳定性能以及更高的安全性。当前，用于合成微流控芯片的聚合物材料主要包括热塑性塑料、固化型材料以及溶剂蒸发型材料等^［26-29］^。固化型材料中的PDMS是一种含有硅基团的弹性液态聚合物，分子主链由［Si（CH_3_）_2_O］ *_n_* 构成（图2），*n*表示硅氧烷链单体的聚合度，在代表硅氧烷链重复次数的同时决定着分子链的长度（*n*数值越大则聚合度越高，分子链越长，分子间的范德华力越强，链与链之间的缠结程度越高，流动性变差，黏度越大，呈半固态；反之，*n*越小则聚合度越低，分子链较短，分子间作用力减弱，缠结少，流动性变好，黏度越小，呈液态）。PDMS具备良好的生物相容性、高柔韧性、强光学透明性、低成本、可抛弃性以及高气体渗透性等特性，这些特性强化了其在生物医学系统中的应用价值，同时也为后续实验创造了良好条件^［30，31］^。PDMS的光学透明性有助于肉眼直接观测芯片微通道内流体的动态变化，同时PDMS对液体的渗透性较低，该特性源于PDMS主链中硅、氧两种原子以共价键结合形成的硅氧（Si-O）键，甲基基团通过硅氧键有序排列形成低能表面，呈现出较强的疏水性，因而适用于微流控芯片的流体控制与样本处理^［32，33］^。此外PDMS具有的高气体渗透性利于需氧型生物反应的检测，但若靶标类型为易挥发小分子时，需要对芯片密封设计进行优化，以减少因靶标挥发导致的损失，确保最终检测结果的准确性。PDMS表面还可实施改性处理，以使PDMS在特定情形下具备独特的应用性能^［34-36］^。此外，PDMS还能够黏附于聚苯乙烯塑料、玻璃等常用基材表面，为后续微流控芯片的制备提供了基础^［37］^。

**图2:**
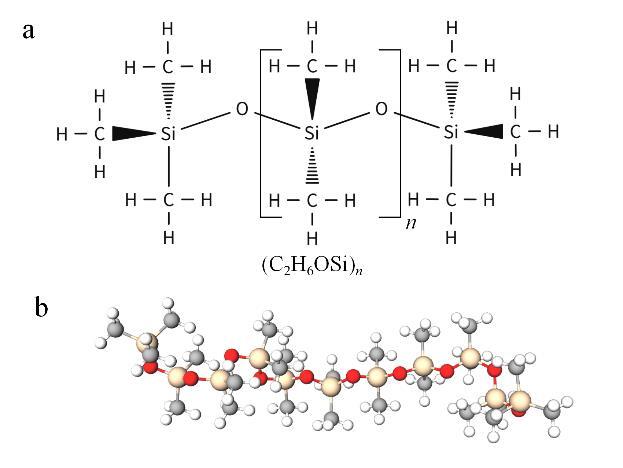
PDMS的结构

微流控芯片根据不同的合成材料，采用相应的制备工艺。PDMS微流控芯片的制备工艺主要包括传统成型技术、新型3D打印技术等^［38，39］^。成型技术工艺简便，其原理是将含有一定体积固化剂的液态PDMS注入带有微通道结构的模具，在85 ℃左右高温环境中干燥固化后拆除模具，获得带有微通道的芯片基板。利用芯片打孔器在微通道的入口与出口处打孔，以保障液体能够进入微通道，最后通过与基底黏合实现封装，整个流程具备环保经济性，适用于芯片的大规模制备^［40，41］^。目前，PDMS微流控芯片通常采用成型技术中的一项微纳成型技术——软光刻技术（soft lithography）制备，同时提供低自发荧光和出色的生物相容性等，便于通过模塑工艺加工成复杂的微通道结构^［42］^。1998年，哈佛大学Whitesides团队^［43］^首次开发基于PDMS的软光刻技术，并迅速成为一种芯片制备的首选方法和工艺。软光刻技术原理为利用化学物质与蚀刻材料表面的化学反应去除多余材料，形成微通道，主要分为涂层、显影、腐蚀和清洁等步骤^［44，45］^。以光刻胶作为材料，实现亚微米级分辨率和精准控制，精准定制曝光剂量与时间以满足所需要求^［46，47］^。Raub等^［48］^采用软光刻技术，借助硅晶片上的SU-8光刻胶主模具进行芯片微成型操作（该芯片的几何结构利用AutoCAD软件进行设计，微流控的流动特性采用COMSOL Muiltiphysics软件进行模拟），最终实现了PDMS微流控芯片的制备流程（SU-8最初用于为半导体器件制造提供高分辨率掩膜，如今主要应用于微流控软光刻技术）。目前，软光刻技术仍是制备微流控芯片的一种标准方法，但该技术实施成本较高、制备过程复杂，需要具备较高的专业技能与丰富的经验，这对芯片的大规模制备可行性构成了挑战^［49］^。为解决这一问题，2024年Yang等^［50］^提出了一种新型硅模工艺，作为SU-8软光刻法的替代方案用于制备PDMS微流控芯片。该工艺运用皮秒（ps）激光切割550 μm厚度的硅晶片，生成最小特征尺寸达50 μm的原始微通道图案；使用KOH溶液修剪边缘碎屑，并通过缓冲氧化物蚀刻（buffered oxide etch， BOE）技术去除保护场氧化层；经阳极键合技术将其固定于玻璃基板上，以用于后续PDMS成型与脱模工艺。此工艺无需光刻设备，为微流控芯片的制备提供了一种新型可替代方案。

除上述经典的成型技术外，3D打印技术近年来也成为制备微流控芯片的一项极具吸引力的技术^［51］^。3D打印技术的原理为运用喷嘴挤出工艺，将塑料、金属等多种材料塑造成三维立体结构。Tony等^［52］^列举了构建PDMS微流控芯片的要求：（1）具备通道的3D结构；（2）拥有微米级的通道与孔洞；（3）保证通道以及孔洞的形状和几何精度（约1~5 μm）；（4）具备适宜的通道表面特征（特别是便于多种PDMS部件的便捷组装或粘接）；（5）可实现大规模生产；（6）具备一定的生产效率和速度。Roh等^［53］^研发了一种运用PDMS开展3D打印的新型高效技术，该技术采用一种毛细管悬浮墨水，此墨水内含有预固化微珠以及未固化液体前驱体形式的PDMS，并以水作为连续介质进行分散。PDMS微珠在液体前驱体诱导的毛细管吸引力作用下相互聚集，形成触变性颗粒糊状。这些毛细管悬浮液具备直接墨水书写所需的高存储模量和屈服应力，可在空气中和水中进行3D打印和固化。经该项技术形成的PDMS结构具备出色的弹性、柔韧性和延展性，可用于打印生物医学产品，或在活组织上直接打印生物支架，在生物医学领域展现出独特优势。Shenoy等^［54］^对利用3D打印模具制备特征尺寸为100~500 μm的芯片进行了研究，提供了打印机校准指南以保障该尺寸范围内的精准打印，并量化分析了打印变化对芯片性能的影响。最终研究结果显示，借助3D打印技术生产的芯片，能够在高度缺陷可变的设备上生成混合效果良好的输出流，证实了3D打印技术适用于低成本芯片的制备。此外还有诸多成功运用3D打印技术制备微流控芯片并将其应用于科学研究的案例^［55-59］^。相较于软光刻技术，3D打印技术在微流控芯片制备领域有望成为一种更为高效且经济的替代方案，用于通过复制成型的方式制备微流控芯片。将3D打印技术与PDMS优良的生物相容性相结合，能够实现高分辨率、快速、可定制化以及一步式制备结构复杂的微流控芯片，在生物医学领域呈现出显著的应用潜力^［60，61］^。尽管3D打印技术在制备PDMS微流控芯片方面具备较大优势，但3D打印机的分辨率、激光光斑尺寸等因素对包括微通道在内的器件结构整体分辨率造成了限制^［62］^。因此，3D打印技术目前尚无法完全或大规模替代光刻技术。

### 1.2 芯片微通道的亲水处理

PDMS材质具备较低的表面张力，可在表面形成均匀的薄膜，针对部分润滑、防水以及防黏附问题呈现出独特的功效。未经处理的PDMS与水的接触角为90°~110°，呈现出疏水性^［63］^。该特性致使PDMS的润湿性能欠佳、流体混合相关问题频发、难以形成均匀的涂层，降低了等离子分离和分子检测等过程的效率。芯片内部液体的传输主要依靠外部泵体的驱动，即泵送效应，以此达成液体从入口向芯片内部的流动，进而完成一系列反应过程，但这种方式在POCT领域具有一定的应用局限性。为解决该问题，研究人员借助聚乙二醇（polyethylene glycol， PEG）［HO（CH_2_CH_2_O） *_n_* H］等亲水物质对PDMS材质芯片的微通道进行修饰。PDMS经清洁活化处理后，先进行PEG连接子（PEG-linker）修饰，随后添加PEG进行进一步修饰，使微通道由原本的疏水性转变为亲水性（图3）。例如Shimada等^［64］^通过PEG一步法对PDMS表面进行了改性处理，经过改性的PEG-PDMS表面在室温环境下储存30天依然保持良好的亲水性。Gonçalves等^［65］^研究并鉴定了块状混合物表面修饰法和表面浸泡法共两种获得亲水性PDMS表面的方法，两种方法均采用了Pluronic^®^ F127、PEG和聚氧化乙烯（polyethylene oxide， PEO）3种不同比例的表面活性剂，通过水接触角（water contact angle， WCA）测量来评估表面润湿性。此外还通过软光刻法采用PEO表面活性剂（2.5%，体积分数）对PDMS进行改性，制备出PDMS微流控芯片。结果证明该方法是使PDMS表面呈现亲水性的最优方法，WCA连续数天低于50°，且对PDMS的光学性能无显著影响。PDMS微通道经亲水修饰后可有效促进液体流动，液体（样本）在亲水性环境下能够借助虹吸效应自发流入微通道，免去了借助外部泵体的推注力（图4），同时拓展了PDMS材料在生物标志物POCT领域的应用。

**图3:**
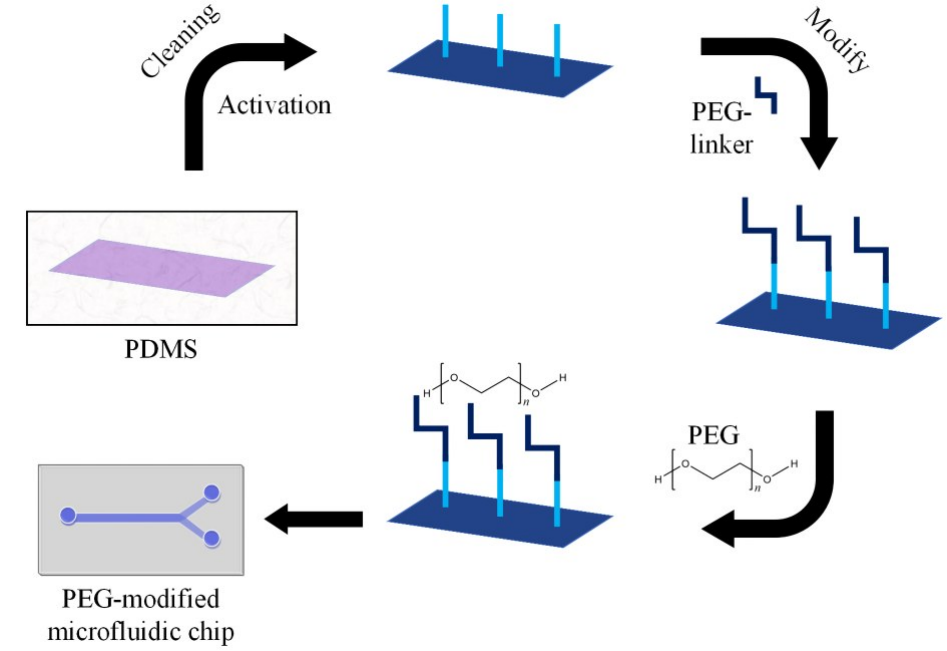
PEG修饰PDMS

**图4:**
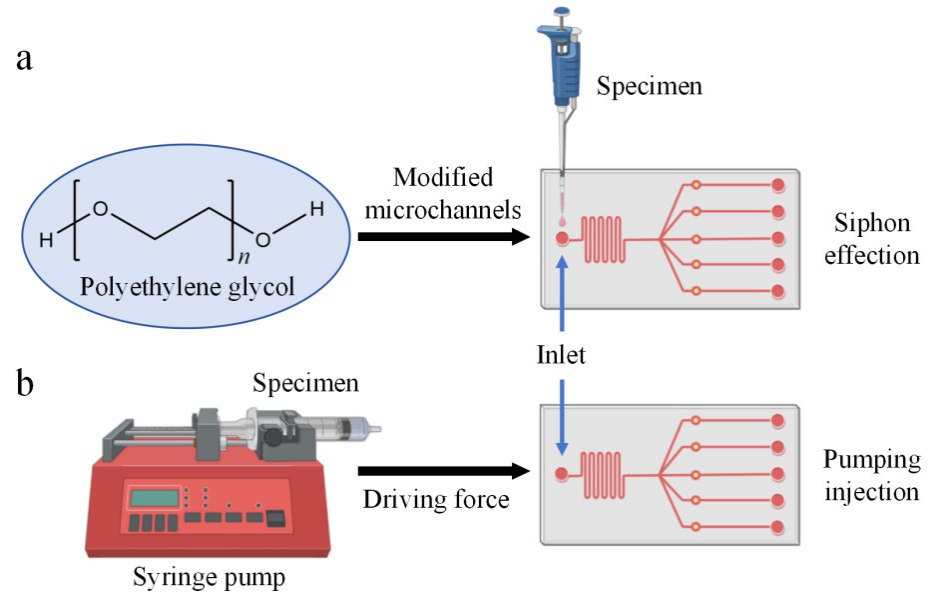
亲水性与疏水性PDMS微流控芯片的区别

PDMS表面除可利用PEG等进行亲水修饰外，还能够采用等离子体处理的方法实施亲水处理^［66］^。等离子体处理属于一种表面改性方法，主要作用是在PDMS表面生成官能团羟基。在等离子体处理过程中，气体于真空环境下发生电离进而形成等离子体，有O_2_、N_2_、NH_3_、H_2_O、CO_2_等多种气体均可用于等离子体处理^［67-69］^。关于PDMS表面氧化的研究多采用真空等离子体处理方法，在用于PDMS等离子体处理的各种气体介质中，O_2_的应用最为普遍^［70，71］^；在此过程中会产生副产物氧自由基，同时氦（He）、氩（Ar）等惰性气体也常被用于PDMS表面的等离子体氧化处理^［72］^。等离子体于放电过程中产生的氧自由基靶向非极性甲基（-Si-CH_3_），以极性官能团硅醇基团（-Si-OH）或氧原子对其进行取代，形成氧化的“类二氧化硅”（SiO *_x_* ）层^［73］^。该基团可将PDMS表面由疏水性转变为亲水性。同时，氧等离子体处理可借助自身的物理作用，在表面改性过程中促使吸附于表面的各类杂质、污染物等发生分解并得以去除，发挥清洁功能^［74］^。等离子体轰击PDMS过程如图5所示。运用等离子体处理方法处理的PDMS表面存在亲水性稳定性欠佳的问题，接触角测量结果显示在暴露20 min内，其疏水性可恢复约40%^［75］^。经PEG处理后的PDMS表面能够维持持久的亲水性。因此，运用化学方法对PDMS表面实施亲水处理，其处理效果的稳定性相较于等离子体处理法有显著提升。

**图5:**
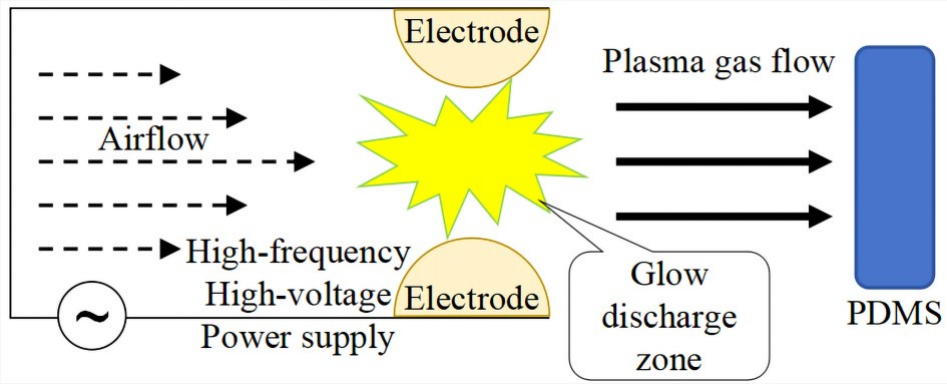
等离子体轰击PDMS材料表面示意图

### 1.3 芯片微通道设计

2004年，Stroock等^［76］^于芯片微通道内首次开发出一种外观呈鱼骨结构的交错人字形混合器（staggered herringbone mixer， SHM），SHM将微流体学与人字形沟槽的独特结构相结合，在微通道内借助液体多方向流动的特性与原理开展液体的被动微混合，发挥静态混合器的作用，并迅速引发了研究人员的关注。SHM可借助光刻与软刻蚀、3D打印以及注塑成型等多种方法与技术进行制备。其中光刻与软刻蚀技术凭借高效率和广泛的应用范围成为主流工艺。该过程包括在硅片或玻璃基底上运用光刻技术构建模板（模具），随后利用PDMS进行微结构的复制和定型。PDMS高保真复制模板图案的特性为此奠定了良好基础，降低了微通道加工的复杂程度。3D打印技术凭借快速原型制作能力适用于复杂结构的制备，但其分辨率存在限制，约为50 μm。注塑成型技术凭借可实现批量生产、适用于塑料材料加工的特性，成为一种重要的工业生产方式。人字形凹凸槽借助周期性扰动流场，诱导横向流动（涡流），打破层流界面，增强对流混合，提升并改善了微通道层流混合效率。2007年，Ansari等^［77］^着重研究了有关沟槽深度和角度的SHM的混合性能、简化及几何优化，经研究发现，液体间的混合受沟槽深度的影响甚于槽角。在SHM结构微通道中，当液体流经沟槽时，因通道截面发生变化而产生离心力，形成旋转涡流（即Dean涡流），促进不同流层间的物质交换，有效增加混合路径，展现出优异性能（图6）。Shen等^［78］^研发了一种结合万古霉素修饰磁珠（vancomycin modified magnetic beads， VMB）的人字形微流控芯片（herringbone-VMB microchip）用于细菌富集，其目的在于促进VMB的均匀分散。结果显示人字形沟槽对液体的流速和流向均具有显著影响，通过人字形沟槽在芯片微通道中引发混沌混合，可提高液体中细菌与固定化磁珠之间的接触概率，实现更高效的细菌富集。Tang等^［79］^提出了一种新型的微流控芯片磁人字形（magnetic herringbone， M-HB）结构，并将其用于检测人获得性免疫缺陷病毒（human immunodeficiency virus， HIV）的互补DNA（complementary DNA， cDNA）序列，以验证该结构在快速灵敏检测方面的优势。相较于传统SHM结构，M-HB结构更易于通过控制磁场实现形成与释放，能够灵敏地检测血样中HIV的cDNA序列，具备灵活性，为进一步深入研究提供了助力。

**图6:**
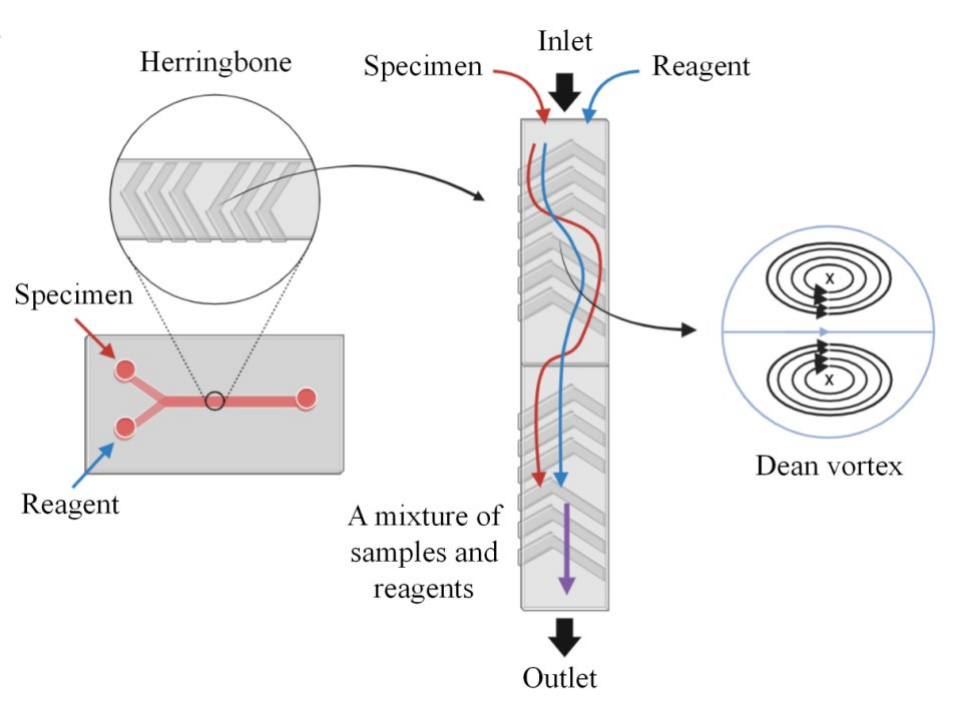
交错人字形混合器及液体产生Dean涡流示意图

## 2 PDMS微流控适配体传感器的开发与处理

PDMS作为合成微流控芯片的常用材料，可借助软刻蚀技术迅速制备复杂的微通道结构，其表面具有较强的可修饰性，便于固定适配体等生物分子。将适配体的高特异性识别能力与PDMS材料优异的微流控特性相结合，在生物标志物检测、疾病诊断、靶向分离等领域具有重要应用价值。修饰的关键在于通过化学或物理方法，将适配体稳定地固定于PDMS微通道内壁，形成一个“识别界面”，实现对样本中靶标的特异性捕获或检测。

### 2.1 PDMS表面预处理

PDMS表面预处理是适配体修饰PDMS芯片的首要且关键步骤，通过引入羟基（-OH）、氨基（-NH_2_）等能够增加表面亲水性的官能团^［80］^，增强与适配体结合的能力，常用方法包括表面等离子体处理、化学蚀刻以及硅烷化修饰^［81，82］^。等离子体处理是最为常用的方法，通过氧等离子体轰击PDMS表面生成羟基，可在数十秒内完成（图7a）^［83］^。化学蚀刻的常用方法是采用由浓H_2_SO_4_与30% H_2_O_2_按7∶3（体积比）组成的食人鱼溶液进行处理（食人鱼溶液具有强腐蚀性与氧化性，操作时需在通风橱中进行并做好防护措施），以提升表面羟基密度（图7b）^［84，85］^。硅烷化修饰则是运用3-氨基丙基三乙氧基硅烷（3-aminopropyl triethoxy silane， APTES）等硅烷化试剂进行处理，引入氨基（图7c），为后续共价结合提供结合位点^［82，86］^。

**图7:**
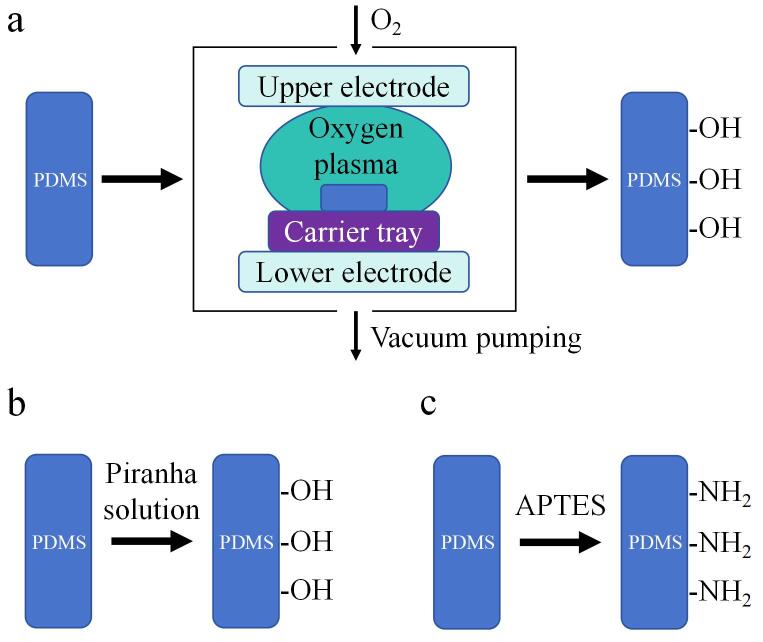
PDMS表面预处理方法

### 2.2 适配体固定化

PDMS表面预处理引入的官能团对固定化方法的选择具有决定性作用。经氨基修饰的表面优先适用于共价固定（例如羧基-氨基酰胺化反应），而经羟基修饰的表面则可辅助非共价静电吸附。适配体固定化步骤需依据PDMS预处理后的表面官能团来选择固定方法，主要分为借助1-乙基-3-（3-二甲氨基丙基）碳化二亚胺（1-（3-dimethylaminopropyl）-3-ethylcarbodiimide， EDC）或*N*-羟基琥珀酰亚胺（*N*-hydroxy succinimide， NHS）接头的共价固定以及通过物理吸附实现的非共价固定^［87］^。共价固定指适配体末端修饰羧基（-COOH）或巯基（-SH）。修饰羧基经EDC或NHS活化后，与PDMS表面的氨基发生反应，形成酰胺键（图8a），实现适配体的共价固定^［88］^；修饰巯基则借助马来酰亚胺（maleimide）或金纳米颗粒（gold nanoparticles， AuNPs）介导与氨基结合，形成二硫（-S-S-）或金硫（Au-S）键（图8b），以此达成共价固定^［89，90］^。非共价固定是借助PDMS表面经等离子体处理后带负电荷的特征，使其与带正电荷的适配体通过静电吸附实现结合（图8c）；生物素修饰的适配体（biotin-modified aptamer， Biotin-Apt）与预先固定于聚二甲基硅氧烷表面的链霉亲和素（streptavidin， SA）相结合（图8d），生物素与SA之间存在的高亲和力（生物素与SA的结合属于当前已知的最强非共价相互作用之一）、结构互补特性（生物素的尿素环结构可完美嵌入SA的结合口袋中的氨基酸残基）以及分子间作用力（氢键、范德华力和疏水性作用）等因素，使得生物素与SA的非共价结合成为生物分子亲和试剂固定与富集过程中不可或缺的策略^［16］^。

**图8:**
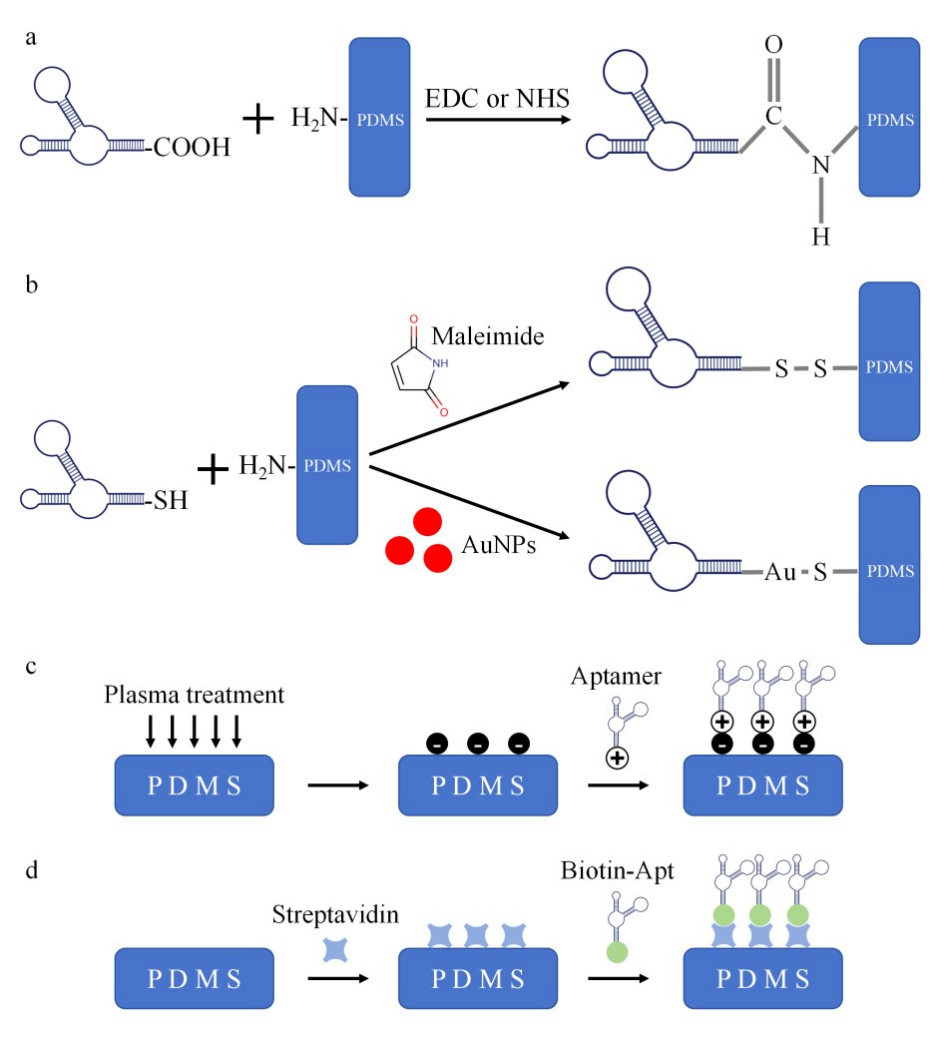
共价固定与非共价固定示意图

共价固定与非共价固定这两种方法各具特性。共价固定具备较高的结合强度与良好的稳定性，能够确保适配体在复杂生物样本中维持牢固状态，不易脱落，以保证检测的准确性与可靠性。但共价固定过程相对繁杂，需要精准把控反应时间、试剂浓度、温度等条件，否则适配体活性可能降低或固定效率欠佳。非共价固定相对简便，无需进行复杂的化学修饰，可在短时间内实现适配体的固定，同时非共价固定中的静电吸附具有一定的可逆性，便于对固定后的适配体更换或调整。然而，此方法的结合强度相对较弱，在样本流速较快或存在较强干扰物质的情形下，适配体可能会从PDMS表面脱落，对最终检测结果造成误差。在实际应用过程中，研究人员需依据具体的检测需求、样本特征以及实验条件等实际状况，综合考量并选取适宜的适配体固定化方法。有时也会将两种固定方法加以结合，以充分发挥二者的特性，提升PDMS微流控适配体传感器的性能与稳定性，更为高效地实现对生物标志物的特异性捕获与检测。

### 2.3 封闭与清洗

封闭与清洗为适配体完成固定化后的最终环节，采用牛血清白蛋白（bovine serum albumin， BSA）、*β*-巯基乙醇（*β*-mercaptoethanol）或其他无关寡核苷酸进行处理，封闭未结合适配体的位点，以降低非特异性吸附。运用磷酸盐缓冲液（phosphate buffered saline， PBS）等缓冲液对微通道实施冲洗操作，去除游离适配体及其他杂质，以保障靶标识别界面的纯净程度。借助精细的封闭与清洗过程，PDMS微流控适配体传感器能够在复杂生物样本中精准捕获并检测靶标，进一步提高其在生物标志物POCT中的准确性与可靠性。

适配体修饰PDMS微流控芯片具备以下4点优势：（1）高特异性：适配体对靶标的识别呈现出分子层面的特异性，与微流控精准的流体操控相结合，能够在复杂样本中实现靶标的高效捕获（如从血液中分离循环肿瘤细胞）；（2）高灵敏度：微通道内部比表面积较大，适配体密度较高，结合微流控快速反应的特性（扩散距离较短），可对皮摩尔至飞摩尔级浓度的靶标进行检测；（3）多功能集成：能够在芯片上集成“样本预处理-适配体捕获-信号检测”的一系列完整流程；（4）低成本与便携性：PDMS芯片制备成本较低，适配体通过化学合成且稳定性较高，适宜开发基于生物标志物POCT的便携式检测设备。适配体修饰的PDMS微流控芯片借助“高特异性识别与微型化流体操控”的协同作用，达成了生物检测的精准化、集成化与便携化，是“芯片实验室”向临床诊断应用转化的关键技术途径。

## 3 PDMS微流控适配体传感器在生物标志物POCT中的应用

微流控技术在基于适配体的检测技术领域呈现出显著的应用潜力。在生物医学检测领域，微流控适配体传感器提供了一种高效、快速且精准的检测方式。传统的生物标志物POCT受限于灵敏度、特异性以及操作复杂性，而微流控适配体传感器的引入则大幅改善或消除了这些弊端。微流控适配体传感器融合了适配体的高特异性与微流控芯片的微型化特点，极大地提升了在微量样本中实现高灵敏度检测的可能性。将适配体固定于微通道内，能够从样本中迅速且有效地捕获靶标，通过优化微流控芯片的设计（如调整通道尺寸、形状及流速），还可进一步提高检测效率与准确性。此外，适配体易于合成和修饰的特性使传感器能够用于检测多种生物标志物，拓展了其在疾病诊断中的应用。

### 3.1 光学微流控适配体传感器

光学适配体传感器作为适配体传感器的典型范例，其对靶标的检测过程依托于光学信号的变化。此类传感器一般运用比色、荧光、化学发光、表面等离子体共振（surface plasmon resonance， SPR）或拉曼散射（Raman scattering）等光学原理，借助适配体与靶标的特异性结合，引发光学信号强度的改变以达到检测目的。光学微流控适配体传感器整合了光学检测的高灵敏度特性与微流控技术的微型化、集成化优势，使在微量样本中开展高灵敏度、高特异性的检测得以实现。

#### 3.1.1 比色微流控适配体传感器

比色适配体传感器作为光学适配体传感器中的经典类别，其检测原理是基于适配体与对应靶标结合引发的颜色变化，以实现可视化检测。该类传感器通常选用金属纳米颗粒作为信号报告分子，对仪器设备的要求不高，可直接通过肉眼判断结果，某些情形下还能借助便携式扫描仪等仪器对结果进行扫描分析，适用于现场POCT。将该类传感器与PDMS微流控芯片相结合，芯片作为传感器的反应平台，在提供微流控操控环境的同时，还可通过固定适配体实现对检测靶标的特异性捕获。例如Zhao课题组^［91］^借助银纳米颗粒（silver nanoparticles， AgNPs）合成了基于PDMS微流控芯片的AgNPs适配体传感器，用于凝血酶（thrombin）的比色检测。将凝血酶与经检测探针适配体Apt 29功能化处理的AgNPs注入微通道，使其与固定于微通道表面的捕获探针适配体Apt 15形成“Apt 15-thrombin-Apt 29-AgNPs”复合物。复合物中AgNPs的含量随凝血酶浓度的升高而增加，引发由黄色至棕色的颜色转变。结果可直接肉眼观察，或借助结合LuxScan 3.0软件的平板扫描仪进行定量分析，特异性较强，对人血清白蛋白（human serum albumin， HSA）、免疫球蛋白A（immunoglobulin A， IgA）、免疫球蛋白G（immunoglobulin G， IgG）等干扰性蛋白无响应。

比色微流控适配体传感器凭借操作简便、成本低廉以及结果直观等优势，在疾病诊断领域展现出广泛的应用前景。此外PDMS微流控芯片的亲水修饰，在促进液体自发流动的同时，还能够减少非特异性吸附，提升检测的准确性。当样本溶液流经固定有适配体的微通道时，适配体捕获靶标与之结合，引发诸如金属离子释放、酶促反应或纳米颗粒聚集等一系列化学反应，导致溶液颜色发生改变。通过比较反应前后的颜色差异，可实现对靶标的定性或半定量检测。

#### 3.1.2 荧光微流控适配体传感器

在光学微流控适配体传感器领域，荧光微流控适配体传感器尤为常见。其检测原理为适配体与靶标结合后，引发荧光信号的增强或淬灭，以此实现检测目的。该类传感器通常以荧光染料或纳米材料为基础制备而成，是一种可实现快速定量检测的高效工具^［92］^。荧光染料属于多环芳烃或杂环烃，在受到激发后能够发射光子，具备快速、灵敏、低成本、易于识别以及生物相容性良好等特性。因而在众多检测及生物成像应用场景中，荧光染料已被广泛应用于获取读出信号。对于生物分子检测而言，理想的荧光染料具备的大斯托克位移（large Stokes shift）至关重要，因其有助于将释放光子的重吸收现象降至最低，可同时提升稳定性和生物相容性^［93］^。相较于其他适配体传感器，荧光适配体传感器凭借其灵活的定量分析能力、高灵敏度、易于应用以及检测范围宽等优势，被广泛应用于生物分子间相互作用的测定^［94］^。PDMS微流控芯片与荧光适配体传感器相结合，在实现样本处理的微量与快速化的同时，还借助精心设计的微通道结构和流体动力学控制，显著提高了检测的精准度。在传感器设计过程中，通过巧妙设计微通道结构，可促进样本中靶标与适配体或其他反应物质的快速混合，加快反应进程。最近，Yu等^［95］^研发了一种用于检测中性粒细胞来源的细胞外囊泡（neutrophil extracellular vesicle， NEV）的中性粒细胞胞外囊泡集成微流控芯片（integrated microfluidic chip for NEV， IMCN）。NEV由于选择性包裹生物活性分子，被视作胃癌液体活检的潜在生物标志物，同时作为特定来源的细胞外囊泡，可降低异质性，提高诊断价值。IMCN为PDMS材质，内部设有SHM结构微通道、磁捕获室和反应室，血清样本与偶联CD66b抗体的磁珠以及CD63适配体进入芯片后，在SHM结构微通道内流动，通过形成复杂涡流增强流体混合，提高碰撞频率，NEV在被CD63适配体标记的同时被磁珠捕获。复合物在磁场作用下于磁捕获室内聚集，使用95 ℃焦碳酸二乙酯（diethyl pyrocarbonate， DEPC）水热裂解富集的NEV，释放CD63适配体和微小核糖核酸（miRNA， miR），并以二者作为引物与3个环状探针（loop probe， LP）进行杂交，在Phi29 DNA聚合酶和脱氧核糖核苷三磷酸（deoxy-ribonucleoside triphosphate， dNTPs）的协助下触发三重滚环扩增（rolling circle amplification， RCA）反应。RCA产物与花青素3（cyanine 3， Cy3）、5-羧基荧光素（5-carboxyfluorescein， FAM）、花青素5（cyanine 5， Cy5）标记的分子信标（molecular beacon， MB）结合，打破MB的荧光淬灭状态，启动变构发夹结构并放大“开启”荧光信号。收集反应溶液并通过细胞成像微孔板检测系统进行测量，进一步运用机器学习算法分析读数以评估诊断性能。IMCN对NEV的捕获率达90%以上，可在4 h内完成10 μL血清样本的分析，为胃癌诊断提供了新型高效的平台。

在设计荧光微流控适配体传感器时，芯片的几何构型亦可采纳其他形状，以优化流体动力学特性与混合效率。为提升传感器性能与检测灵敏度，微流控芯片设计可融入创新性的液体混合策略，如分流、合流、旋转及振荡等。通过精准操控液体流动与混合过程，能够实现对目标分析物的快速且精准的检测。基于这些创新性设计与混合技术，可研发出一系列新型荧光微流控适配体传感器，以满足不同应用领域的需求。例如Liu等^［96］^设计了一款圆形旋转荧光阵列微流控芯片，该芯片采用PDMS-玻璃双层结构材质，包括3个独立检测区域，每个区域含有一对AuNPs金盘，其中左盘固定由不完全互补链fDNA与适配体杂交形成的双链，右盘固定与适配体完全互补的单链cDNA，均通过Au-S键进行固定，用于同时检测金黄色葡萄球菌（*S. aureus*， *S.A*）、鼠伤寒沙门氏菌（*S. typhimurium*， *S.T*）和副溶血性弧菌（*V. parahemolyticus*， *V.P*）。当样本加入芯片时，靶标与左盘适配体相结合，释放出适配体-靶标复合物，复合物随500 r/min的转速旋转并流向右盘，适配体与右盘的cDNA结合形成dsDNA。加入SYBR GreenⅠ染料后，染料嵌入dsDNA中产生荧光，同时病原体被释放回左盘，实现信号的循环放大。通过智能手机连接自制黑盒，采集荧光信号（绿色通道G值），并以右盘与左盘荧光强度比（G_r_/G_l_）进行定量分析。该芯片凭借循环信号放大、无泵旋转驱动、比率定量这三大核心创新点，有效解决了传统微流控芯片“依赖设备、操作复杂、抗干扰差”的问题，30 min内可完成多靶标同步检测，各检测区相互独立，可避免交叉干扰，充分彰显了其在现场POCT方面的应用潜力。

#### 3.1.3 化学发光微流控适配体传感器

化学发光（chemiluminescence， CL）适配体传感器借助适配体与靶标结合触发化学发光反应以实现检测。化学发光反应一般源于发光物质在化学反应中从激发态回归基态时释放光子。该类传感器通常依托鲁米诺（luminol）、过氧化草酸酯（peroxyoxalate， PO）或高锰酸钾（potassium permanganate， KMnO_4_）等化学发光试剂，这些试剂在与适配体结合的靶标或其他反应物相互作用下被激活，进而产生化学发光信号。PDMS微流控芯片与化学发光适配体传感器相结合，进一步提升了检测的微型化、集成化与自动化水平。微流控芯片营造的微环境有助于调控化学反应条件，如温度、pH值以及反应时间等，优化了化学发光反应的效率。与此同时，通过对微通道形状进行特殊设计，能够实现样本的精准操控与高效混合，进而提高检测速度与准确性。适配体被固定于微通道内部，当样本流经微通道时，适配体与其中的靶标相结合，触发化学发光反应，产生的光信号在被光电探测器捕获的同时转换为电信号，经过数据处理与分析便可实现对靶标的定量检测。该类传感器无需外部光源，且具有背景信号低、灵敏度高的特点。Peng等^［97］^研发了一种基于微流控化学发光的生物传感器，结合杂交链式反应（hybridization chain reaction， HCR）信号放大技术，实现外泌体miRNA（miR）-21与155的高灵敏多重检测。该传感器采用PDMS材质，运用软光刻与复制成型技术制备而成，包含蛇形混合通道、外泌体捕获通道、检测通道共3个功能单元。蛇形混合通道用于充分混合鲁米诺与H_2_O_2_，为CL提供均匀试剂；外泌体捕获通道含有Y形阵列，通道表面经氨基化处理、交联剂活化后，偶联用于捕获外泌体的CD63-Apt或发夹探针，并封闭非特异性位点，同时Y形阵列可提高捕获效率；检测通道由2条平行的船形通道构成，生物素标记的发夹探针可实现HCR扩增与CL检测（采用BPCL-2超微弱发光测量仪进行监测），能够同步检测miR-21和155，为乳腺癌的早期诊断提供了一项精准的检测工具。

#### 3.1.4 拉曼散射微流控适配体传感器

拉曼散射适配体传感器依托拉曼散射效应，借助适配体与靶标结合后引发的拉曼光谱变化开展检测工作。此类传感器具备指纹识别特征，能够实现对靶标的高特异性检测。与表面增强拉曼散射（surface-enhanced Raman scattering， SERS）技术相结合，可进一步提升拉曼散射传感器的灵敏度。Sheng等^［98］^研制出一种基于SERS的PDMS材质微流控芯片，该芯片结合识别竞争策略与磁聚集双信号放大技术，实现了对肝癌相关蛋白（肿瘤标志物）中甲胎蛋白（alpha fetoprotein， AFP）和锰超氧化物歧化酶（MnSOD）的双靶标快速同步检测。在芯片制备过程中经PEG处理以使其具备亲水性，样本于亲水微通道内借助虹吸效应自发流动且无泄漏现象，通过适配体互补配对形成检测复合物“Raman signal molecule@AuNPs@H-Fe_3_O_4_@cDNA”，靶蛋白与适配体的特异性结合会竞争性置换该复合物，致使SERS信号降低。反应时间仅5 min，在裸鼠肝癌模型血清中的检测结果与酶联免疫吸附测定（ELISA）结果相符，为肝癌早期诊断提供了一种高效的方法。

SERS技术与微流控芯片的融合在研发新型适配体传感器的同时也为研制可直接与皮肤接触的微流控芯片创造了条件。该技术可使皮肤表面分泌的汗液经微孔道直接流入芯片，并于芯片内对特定生物标志物进行检测，省去了借助移液器或泵送等中间环节。在特定应用场景下，还能够设计集成式手动阀门，以实现对液体流动和检测过程的精准把控。例如Yang等^［99］^研制出一种PDMS材质的可穿戴SERS微流控芯片，借助手动操控阀门达成汗液中尿素（urea）和尿酸（uric acid， UA）的双靶标实时监测。该芯片以AgNPs单层作为SERS基底，结合5-FAM荧光标记的适配体对靶标进行特异性捕获。采用632.8 nm激光（0.5 mW）激发，积分时间为5 s，通过监测1 325 cm^-1^处的SERS信号变化开展定量分析，信号强度与靶标浓度呈负相关。在志愿者实验中，该芯片的检测速度快于传统临床血液检测，准确性与之相当（两种靶标的血液浓度估算值与临床检测结果偏差较小），为肾脏健康的无创实时监测提供了一种新手段。

光学微流控适配体传感器凭借其高灵敏度、强特异性以及微型化、集成化等技术优势，在生物标志物POCT中被广泛用于肿瘤标志物检测、病原菌检测等领域。然而，截至目前该类型传感器在微通道设计、材质选择等方面仍存在一定的局限性。随着新型光学材料、光学检测技术的不断涌现以及微流控技术的持续进步，光学微流控适配体传感器将在生物标志物POCT领域发挥日益重要的作用。

### 3.2 电化学微流控适配体传感器

在经典适配体传感器领域，电化学适配体传感器具备较高的灵敏度与选择性。其工作机制建立于适配体与靶标结合后引发的电化学信号改变，可借助电化学方法予以检测，具有设备简易、便于微型化与集成化以及成本较低等特性。将微流控芯片与该类传感器相结合，能够开发出基于电化学的微流控适配体传感器，即电化学微流控适配体传感器。电化学微流控适配体传感器融合了电化学传感与微流控技术的特点，可实现对生物标志物的快速、灵敏检测，为生物标志物的POCT提供了一种高效、精准的方法。在开发基于PDMS材质微流控芯片的电化学微流控适配体传感器时，鉴于PDMS的高分子结构能够形成一种绝缘层，其具备良好的电绝缘性能，该性能为电化学适配体传感器的研发提供了有力支撑，可使电化学信号更为稳定，提升了检测的准确性。此外，PDMS的可塑性与易加工性为电化学微流控适配体传感器的设计赋予了高度的灵活性，能够依据靶标检测的实际需求对微通道结构进行定制，优化传感器的性能。此外，借助PDMS的微加工技术，能够制备出具备复杂三维结构的微流控芯片，为电化学适配体传感器的创新性设计提供了更多的可能性。Siavash等^［100］^研发了一种基于适配体的电化学微流控生物传感器，该传感器融合3D金纳米/微米岛（nano/micro-islands， NMIs）与微流控技术，以达成微小隐孢子虫（*C. parvum*）卵囊的快速检测。微流控芯片与可拆卸吸引按钮均由PDMS合成，芯片借助负压驱动达成样本的自动传输，规避交叉污染。其检测机制为卵囊与对应的适配体相结合形成复合物，在电极表面产生空间位阻效应，阻碍氧化还原探针（［Fe（CN）_6_］^3-/4-^）的电子转移以及向电极表面的扩散，降低电子传递效率，致使通过差分脉冲伏安法（differential pulse voltammetry， DPV）测得的峰电流数值降低。另外该传感器具备极佳的稳定性，冻干电极在室温条件下储存21天仍能保持85%的活性。该传感器凭借3D金NMIs的高比表面积以及适配体的高特异性，实现了*C. parvum*卵囊的超灵敏检测，未来集成智能手机电化学检测模块有望实现全自动化POCT设备的研发。

电化学微流控适配体传感器能够与其他信号放大策略相结合，进一步提升检测灵敏度（如借助酶催化反应、纳米材料放大效应等）。电化学微流控适配体传感器在疾病生物标志物POCT领域展现出广泛的应用前景，通过融合微流控技术的微型化、集成化特性以及电化学传感的高灵敏度，可达成对生物标志物的快速、精准检测。

### 3.3 双模态微流控适配体传感器

自微流控适配体传感器诞生以来，其研究进展与应用实践经历了比色法、荧光法、化学发光法及电化学法等多个发展阶段。在实际应用中，这类单一检测模式的传感器通常呈现出一定的局限性，例如检测手段单一、易出现假阳性或假阴性结果等问题。随着科技的发展，融合两种不同检测技术的双模态微流控适配体传感器应运而生，在双重检测模式的支持下，检测灵敏度得以显著提高，并在生物标志物POCT领域获得广泛应用，取得了显著的检测成效。在PDMS微流控适配体传感器的研发与应用过程中，同样涉及该方法与技术的融合。将两种不同类型的检测技术相结合，可发挥二者的互补优势，在一定程度上降低检测结果的误差。例如Lin等^［101］^构建了一种PDMS微流控芯片与光电化学-荧光双模态传感器相结合的检测体系。以循环肿瘤细胞（circulating tumor cells， CTCs）中的非小细胞肺癌（non-small cell lung cancer， NSCLC）NCI-H460和NCI-H1650细胞为研究模型，借助微流控芯片对全血中经同型半胱氨酸-硫醇（Hcy-thiol）探针标记的CTCs进行分离。利用基于RA16和EGFR两种适配体修饰的CuInS_2_纳米花构建阴极光电化学传感系统，该系统可选择性捕获CTCs（RA16用于捕获NCI-H460细胞，EGFR用于捕获NCI-H1650细胞）并提供光电流信号，实现双模态检测。在优化条件下，CTCs的分离效率和纯度分别达到83.4%和76.8%，在100 cells/mL的加标血样中，通过光电流响应测得NCI-H460细胞浓度为（107±5） cells/mL，NCI-H1650细胞浓度为（87±4） cells/mL，荧光信号验证结果分别为（110±4）和（85±3） cells/mL，该研究为NSCLC早期检测和转移评估提供了一种具有高特异性与高灵敏度的检测工具。

在双模态微流控适配体传感器的研发进程中，对微流控芯片的微通道结构开展特殊设计，能够发挥高效捕获与分离的功效，为靶标的精准检测营造良好的环境。例如Chen等^［102］^研发出一种集成双模态的PDMS微流控芯片，将近红外光电化学（photoelectrochemical， PEC）适配体传感器与荧光成像技术相结合，从全血样本中高效分离HepG2与Hep3B两种肝癌CTCs并实施表型分析。该芯片的微通道运用光刻技术制造出SHM结构（内含2个丝网印刷电极），基于Yb-Bi_2_S_3_@AuNPs纳米复合材料构建PEC传感系统，通过CD133与GPC3双适配体功能化表面特异性捕获两种靶细胞，借助“轨道交通”原理基于空间效应筛选不同靶细胞表型。该原理为引入芯片的不同肝癌CTCs依据表面标志物被不同适配体选择性捕获：CD133适配体区域捕获Hep3B细胞，GPC3适配体区域捕获HepG2细胞。采用半花菁荧光探针标记靶细胞以进行荧光成像，CTCs分离效率达到90%。该芯片兼具PEC的高灵敏度和荧光成像的准确性（PEC近红外激发，可实现高灵敏度定量；荧光成像能实时进行空间确认，减少假阳性和假阴性），达成了CTCs的高特异性表型筛选与定量分析，为肝癌等恶性肿瘤的早期诊断、治疗监测等提供了支撑。

尽管双模态微流控适配体传感器在检测精准度、灵敏度等性能层面具有显著优势，但也不可避免地存在一些关键局限。例如两种检测技术可能出现信号相互干扰的情况，这可能会对最终检测结果造成影响。此外，双模态微流控适配体传感器的集成工艺相对复杂，增加了制造成本和制造难度。因此，为进一步优化双模态微流控适配体传感器的性能，研究人员需重点解决如下问题：例如可通过改进传感器设计，减少两种检测技术间的信号串扰；探索更为简便、高效的集成工艺，以降低制造成本和难度。这些优化措施将为双模态微流控适配体传感器的后续技术研发提供重要的方向和参考。

PDMS微流控适配体传感器在生物标志物POCT领域已取得显著成效。例如在癌症早期诊断过程中，PDMS微流控芯片可借助适配体对肿瘤细胞或某些蛋白类肿瘤标志物的特异性识别功能，实现对靶标的快速捕获与检测。通过与荧光标记或其他信号放大技术相结合，能够进一步提升检测的灵敏度与准确性。此外，PDMS微流控适配体传感器还在传染病病原体检测、多种生物标志物监测等多个领域得到应用，呈现出广阔的应用前景。为使讨论更为完善，表1中展示了基于微流控技术的不同适配体传感器在生物标志物检测中的性能对比情况。值得注意的是，基于微流控的电化学适配传感器在检测生物标志物时，其线性范围和检出限性能明显优于光学检测技术。

**表1:** 基于PDMS微流控芯片的生物标志物适配体传感器的性能指标

Detection method	Strategies of specificity	Targets	Aptamer modifications	Linear range	LOD	Ref.
Colorimetric	visual inspection or quantitative analysis by scanner	thrombin	AgNPs	20-5000 pmol/L	20 pmol/L	［^91^］
Fluorescence	measured using a cell imaging microplate detection system	NEV released miR	/	10^5^-10^9^ EVs/mL	miR-223-3p： 0.61 fmol/L； miR-425-5p： 0.24 fmol/L	［^95^］
	collecting fluorescence signals for quantitative analysis	*S.A*， *S.T*， *V.P*	AuNPs	*S.A* and *S.T*： 10^2^-10^8^ CFU/mL；*V.P*： 10^1^-10^8^ CFU/mL	*S.A*： 36 CFU/mL； *S.T*： 39 CFU/mL， *V.P*： 7 CFU/mL	［^96^］
Chemi-luminescence	biotin-labeled hairpin probes enable HCR amplification and detection	miR-21， miR-155	/	1 fmol/L-10 pmol/L	miR-21： 0.39 fmol/L； miR-155： 0.49 fmol/L	［^97^］
Raman scattering	rectangular detection zone via magnetic aggregation concentration detection	AFP， MnSOD	4-MBA， DTNB	10^-12^-10^-6^ g/mL	AFP： 5.89 pg/mL； MnSOD： 6.23 pg/mL	［^98^］
	SHM promotes hybridization， enabling signal readout via nanoprobes	prostate cancer cell exosomes	AuNPs	1-10^5^ particles/μL	1 particle/μL	［^103^］
	target binding to the aptamer replaced FAM， resulting in signal attenuation	urea and UA	5′-FAM	/	/	［^99^］
Electro-chemistry	target binding to the aptamer impedes electron transfer	*C. parvum* oocysts	/	10-10000 oocysts/mL	10 oocysts/mL	［^100^］
	differential pulse voltammetry	cortisol hormone	/	1 pmol/L-1 μmol/L	0.2 pmol/L	［^104^］
Dual-modal	photoelectrochemistry and fluorescence	NSCLC cells NCI-H460， NCI-H1650	AuNPs	NCI-H460： 50-1×10^6^ cells/mL； NCI-H1650： 50-5×10^5^ cells/mL	NCI-H460： 16 cells/mL； NCI-H1650： 15 cells/mL	［^101^］
	near-infrared photoelectrochemical and fluorescence imaging with supplementary SHM	liver cancer CTCs cells HepG2 and Hep3B	AuNPs	100-5×10^6^ cells/mL	HepG2： 8 cells/mL； Hep3B： 6 cells/mL	［^102^］

AgNPs： silver nanoparticles； NEV： neutrophil extracellular vesicle； EV： extracellular vesticle； *S.A*： *Staphylococcus aureus*； *S.T*： *Salmonella typhimurium*； *V.P*： *Vibrio parahaemolyticus*； AuNPs： gold nanoparticles； CFU： colony forming units； HCR： hybridization chain reaction； AFP： alphafetoprotein； MnSOD： manganese superoxide dismutase； 4-MBA： 4-mercaptopropanolic acid； DTNB： 5，5′-dithiobis-（2-nitrobenzoic acid）； SHM： staggered herringbone mixer； FAM： carboxyfluorescein； UA： uric acid； NSCLC： non-small cell lung cancer； CTCs： circulating tumor cells.

## 4 总结与展望

伴随科学技术的持续进步，基于适配体的生物传感技术不断推陈出新，以微流控适配体传感器为典型范例的检测技术在生物标志物POCT领域已展现出巨大的应用潜力与价值。适配体传感器依托适配体特异性识别靶标的生物分子相互作用机理，同时结合传感器技术将该作用转化为电信号、光信号或质量变化等可量化的物理信号，最终达成对靶标的定性与定量分析。鉴于适配体在温度稳定性方面的特征，适配体传感器适用于在气候温暖或炎热的偏远地区开展生物标志物POCT，以及在流行病暴发期间对潜在感染进行大规模筛查^［105］^。在微流控适配体传感器领域，具有疏水性本质的PDMS经亲水性修饰后制备的微流控芯片可借助虹吸效应引导液体直接流入微通道。该类芯片无需额外配备泵送设备即可实现快速检测，具备显著的便捷性，且对检测结果无不良影响，为POCT领域提供了一种具有高度可操作性的方法。PDMS微流控芯片在提供精准的流体操控平台的同时，还通过适配体的固定化增强了对靶标的捕获能力。

相较于传统适配体传感器，微流控适配体传感器借助微通道的精准设计，达成对样品的精准操控以及靶标的处理，能够在短时间内完成复杂的检测流程，呈现出新兴技术的特征。与此同时，该技术也面临着一些挑战与问题，例如需进一步简化操作流程、降低生产成本、提高传感器稳定性以及在复杂样本中实现更精准的检测等。为促进微流控适配体传感器在生物医学检测领域的广泛应用，后续需持续探索适配体的筛选与传感器合成技术，从而进一步提高适配体传感器对靶标的亲和力与特异性；优化微流控芯片的设计与制造流程，以实现更高效、更精准的液体操控与检测；深入探究不同检测技术的融合与互补性，大力研发具备双重模式检测功能的双模态微流控适配体传感器以及可一次检测多种靶标的微流控适配体传感器，进而增强检测的准确性、可靠性以及传感器的实用性。此外，传感器的微型化与集成化亦是未来的关键发展趋向，旨在达成更高效的样本处理以及更快速的检测结果输出。为应对这些挑战，科研人员需持续探寻多种新型纳米材料、高分子材料以及3D打印技术等新途径，以促进微流控适配体传感器的技术创新与应用拓展，并将研发所得的新型传感器充分运用于生物标志物POCT领域，为疾病诊断等提供高效便捷的创新性手段。
